# HIV/AIDS Vaccine: An Update

**DOI:** 10.4103/0970-0218.66862

**Published:** 2010-04

**Authors:** Anita Nath

**Affiliations:** Population Council, 1230 York Avenue, New York City, NY-10065, United States

## Introduction

The global HIV/AIDS epidemic continues to take its toll of human lives. In 2007 there were 33 million people living with HIV/AIDS, with 2.7 million new HIV infections and two million HIV-related deaths.(1) With a total of 2.5 million people living with HIV/AIDS, India too is in the grip of the HIV/AIDS epidemic.(2) According to the HIV Sentinel Surveillance data 2004–2006, there are 156 Category A districts (where there is more than one per cent antenatal prevalence at any given time, in any of the sites, in the last three years).(3) Despite the recent progress in increasing access to treatment and prevention programs, the epidemic continues to outpace the global prevention efforts. In countries that are most heavily affected, the epidemic has led to a significant increase in household poverty and reduction in the life expectancy by more than 20 years.(4,5) The effects of gender inequality leave women and girls more at risk of exposure to HIV. In India, women are becoming increasingly vulnerable to HIV/AIDS and account for around one million cases.(6) Moreover, they have fewer and rather unfeasible options available to protect themselves such as abstinence and use of condom. The best hope of ending the epidemic lies in the development of a suitable vaccine.(7) Vaccines are one of the most effective public health interventions ever known. An HIV vaccine could either prevent disease onset or progression to AIDS. According to mathematical estimates by the International AIDS Vaccine Initiative (IAVI) - provided that other programs for treatment and prevention have been scaled up — an HIV vaccine with an efficacy as low as 30% and coverage as low as 20% could avert as many as 5.5 million new infections between the years 2015 and 2030.(8) The development of an effective vaccine has posed a wide range of challenges, as HIV has proven to be a uniquely complex virus.

## Types of HIV Vaccines

Broadly speaking, the potential HIV vaccines may be classified as:(9)

Live attenuated vaccines: Although scientists have studied live attenuated vaccines against HIV in animals, where they have shown high levels of protection, they are not currently being developed for use in humans because of safety concerns.

Subunit vaccines: Subunit vaccines contain a small protein or piece of the pathogen; the protein acts as a foreign antigen and elicits B cells of the immune system to produce antibodies against the antigen. The first AIDS vaccine developed and tested was designed using the subunit concept. The first AIDS vaccine to go through complete testing in humans, the AIDSVAX gp120 vaccine, was a subunit vaccine. This vaccine failed to protect against HIV infection in an efficacy trial, which is one of the reasons why scientists are working to discover better vaccine concepts.

DNA vaccines: This vaccine entails use of copies of single or multiple genes from the pathogen. The genes from the pathogen integrate with the human gene resulting in the formation of a protein, which is seen as a foreign or harmful antigen by the immune system and results in the production of an immune response. This is a common strategy being used for AIDS vaccine development, and many of the current AIDS vaccine candidates are DNA vaccines. DNA vaccines will not cause HIV infection, because the vaccines do not contain all the genes of the live pathogen.

Recombinant vector vaccines: This adopts the same strategy as DNA vaccines, except that the genes are carried by a harmless or a much weakened bacterium or virus, called a vector. Many of the current AIDS vaccine candidates are vector vaccines. Recombinant vector vaccines will not cause HIV infection because they contain copies of only one or several HIV genes, not all of them. Many scientists believe that the addition of a vector will allow the vaccine to be more effective in creating an immune response, rather than a DNA vaccine alone. Some of the candidate recombinant virus vectors include adeno and adeno-associated viruses, pox viruses such as Modified Vaccinia Ankara (MVA), and alphaviruses such as Venezuelan Equine Encephalitis (VEE) virus.

## Clinical Trial Phases for a Candidate Vaccine

Broadly speaking, the clinical trial of a candidate vaccine is divided into three phases:(10)

- Phase I: Phase I trials are conducted on small numbers (10-30) of healthy adult volunteers who are not at risk of HIV, to evaluate the safety and perhaps analyze the immune responses evoked by the vaccine, of different vaccine doses and immunization schedules. A Phase I trial usually takes 8-12 months to complete.
- Phase II: Phase II testing generates additional safety data as well as information for refining the dosage and immunization schedule. It involves a larger number of volunteers (50-500), which include a mixture of low-risk people and higher-risk individuals. These trials generally take 18-24 months to complete. The efficacy of the candidate vaccine may be tested in a version of this trial known as Phase IIb trial, also called ‘test of concept trial’ or step study. These trials enable the researchers to decide if the candidate is worth testing in larger Phase III trials. Hence, it helps to avoid unnecessary wastage of resources.
- Phase III: Phase III trials are conducted to assess vaccine efficacy. It involves thousands of volunteers from high-risk populations. The minimum duration of a Phase III trial could go up to a period of three years.

## Major Challenges Toward the Development of an Effective HIV/AIDS Vaccine

Although it has been decades since the onset of the HIV epidemic, the wait is still on for the discovery of an effective vaccine.

- Scientific challenges: The nature of the HIV virus has created a number of obstacles in the way of development of a potent HIV/AIDS vaccine. These include:
  - Genetic diversity: Once the HIV virus enters the CD4 cell, it multiplies at a very rapid pace resulting in the production of copies that are genetically diverse from the parent virus. This makes development of an effective AIDS vaccine much more difficult because it will have to protect against so many different virus strains.(11)
  - Formation of neutralizing antibodies: None of the vaccine candidates have been effective in inducing the formation of neutralizing antibody against the globally diverse and circulating strains of HIV. Most licensed vaccines against other diseases are thought to work because they induce virusspecific neutralizing antibodies.(12) Moreover, there is lack of information on the immunological correlates of protection against HIV/AIDS.(13)
  - Lack of a proper animal model for pre-clinical testing: Prior to testing in humans, vaccine candidates are developed and tested extensively in the laboratory and then in different animal models. In relation to the development of the HIV vaccine, the vaccine candidate is administered to macaques that are later infected with simian immunodeficiency virus (SIV), because HIV does not infect any other animal.(14) As a result, the animal model for testing the vaccine is not proper as it cannot mimic the actual HIV infection, as in humans.
- Programmatic challenges:(15–17)
  - HIV vaccine clinical trials are difficult, long, and expensive.
  - Insufficient funding allocated to vaccine development.
  - Recruiting volunteers may be difficult in developing countries because of less education, myths, and misconceptions about the vaccine.
  - Lack of political commitment.
  - Slow approval processes in developing countries.

## Ongoing Global Clinical Trials

A number of institutions are involved in HIV vaccine development, especially the US National Institutes of Health, the US Military HIV Research Program, the US Centers for Disease Control and Prevention, the French National Agency for Research on AIDS, the European Community, the International AIDS Vaccine Initiative, WHO, UNAIDS, and others, including the vaccine industry. The first phase I trial of an HIV candidate vaccine was conducted in USA in 1987, using a gp160 candidate vaccine. At present there are 24 candidate vaccines in various stages of development.(18) Out of these, 18 are undergoing the Phase I trial, three are in Phase I/II trials, two are in the Phase II trial, and only one is in the Phase III trial; which is VaxGen’s gp120-based AIDSVAX, which is being tested in Thailand. The trials are ongoing in many countries in the developing world, including Botswana, Haiti, Kenya, Peru, South Africa, and Uganda. Table 1 shows the types of HIV/AIDS vaccine in different stages of development.

**Table 1:** Ongoing clinical trials

Phase	Candidate vaccine	Vaccine type	Number of volunteers
Phase I	Ad26.EnvA-01	Viral vector - Adeno	48
	Ad5HVR48.ENVA.01	Viral vector - Adeno	48
	HIVIS-DNA/MVA-CMDR	DNA/Viral vector - Pox	24
	ALVAC-HIV vCP1521	Viral vector - Pox	50
	Vichrepol	Protein	15
	PENNVAX-B	DNA	120
	SAAVI DNA-C2/SAAVI MVA-C	DNA/Viral vector - Pox	120
	VRC-HIVADV027-00-VP/VRC-HIVADV038-00-VP/VRCHIVDNA044-00-VP	Viral vector - Adeno/Viral vector -Adeno/DNA	192
	Ad35-GRIN/ENV/Ad35-GRIN/ENV	Viral vector - Adeno/Viral vector - Adeno	42
	ADVAX	DNA	40
	ADVAX/TBC-M4	DNA/Viral vector - Pox	32
	ALVAC-HIV MN120TMG strain (vCP205)	Viral vector - Pox	36
	VRC-HIVDNA009-00-VP/VRC-HIVADV014-00-VP	DNA/Viral vector - Adeno	29
	MVA-CMDR	Viral vector - Pox	48
	Chinese DNA /Tiantian vaccinia	DNA/Viral vector - Pox	80
	VRC-HIVDNA016-00-VP/VRC-HIVADV014-00-VP	DNA/Viral vector - Adeno	60
	VRC-HIVADV027-00-VP/VRC-HIVADV038-00-VP	Viral vector - Adeno/Viral vector - Adeno	35
	VRC-HIVADV014-00-VP	Viral vector - Adeno	40
Phase I/II	pHIS-HIV-AE/rFPV-HIV-AE	DNA/Viral vector - Pox	8
	HIVIS-DNA/MVA-CMDR	DNA/Viral vector - Pox	60
	DNA-C/NYVAC-C	DNA/Viral vector - Pox	147
Phase II	pGA2/JS7 DNA/MVA/HIV62	DNA/Viral vector - Pox	225
	VRC-HIVDNA016-00-VP/VRC-HIVADV014-00-VP	DNA/Viral vector - Pox	225
Phase III	ALVAC-HIV vCP1521/AIDSVAX gp120 B/E	Viral vector - Pox/Protein	16,403

## Clinical Trials in India

In India, leading institutions including the Indian Council of Medical Research (ICMR), the National AIDS Control Organization (NACO), and their partner International AIDS Vaccine Initiative (IAVI), and institutions under the Department of Biotechnology (DBT) have been intensively involved in the area of AIDS vaccine development. The Indian HIV vaccine development program has always been focusing on the development of vaccines designed to prevent HIV subtype C.(15,26) The first ever conducted Phase I trial in India was that of an adeno-associated virus (AAV)-based vaccine (tgAAC09) expressing *gag, protease, delta*-RT HIV-1 subtype C genes, administered intramuscularly to 30 healthy, HIV-uninfected male and female adult volunteers. It was initiated in February 2005, at the National AIDS Research Institute (NARI), Pune, as part of a joint trial with centers in Germany and Belgium, and was completed successfully in December 2006. It was observed that the vaccine was generally safe, well-tolerated, and modestly immunogenic.(20,21) Another Phase I trial had been initiated in Chennai, in January 2006, to assess the safety and Immunogenicity of Modified Vaccinia Ankara (MVA)-based vaccine (TBC-M4), an attenuated form of the vaccinia virus expressing *env, gag, tat-rev*, and *nef-RT* HIV-1 subtype C genes.(22) Preliminary results of this trial have suggested safety, tolerability, and 100% immunogenicity.(23)

## Conclusion

There is an urgent need for a safe, effective, affordable, and feasible HIV/AIDS vaccine, to counter the rapidly progressing epidemic. An HIV vaccine with an efficacy threshold of as low as 30% efficacy has been suggested to be acceptable and likely to be taken up by paying individuals in the private market.(24) Conducting AIDS vaccine trials, especially in countries like India requires cooperation and coordination from different segments of society, which include political leaders, program managers, policy makers, scientific communities, non-governmental organizations, community leaders, and HIV-affected populations. Critical ethical issues would need to be handled when conducting these trials, including the level of care and treatment that should be offered to the volunteers, who may become infected during the course of the trial.(25) Involvement of public health specialists in vaccine development is of paramount importance, so that research findings are rapidly transformed into effective and acceptable programs.
